# The Military Service Acts and the Profession

**Published:** 1916-06-17

**Authors:** 


### The Hospital

The Workers' Newspaper of Administrative Medicine and Institutional Life, Administration, National Insurance and Health.

No. 1566, Vol. LX. SATURDAY, JUNE 17, 1916. PRICE ONE PENNY.

### THE MILITARY SERVICE ACTS AND THE PROFESSION.

The new Military Service Act has an urgent and important relation for all British medical men who have not attained the age of forty-one years before the 24th of the present month. On that date every such practitioner, whether married or unmarried, who has not applied for and obtained exemption will be " deemed " to have enlisted for military service; and in due course, and with the other members of the " class " in which his age places him, he will be called upon to discharge the obligation thus defined. When this event arrives, though it is possible that the authorities might pass the practitioner for general combatant service, the probability is that he would be offered a commission in the R.A.M.C. In these latter circumstances service would be for the duration of the war and the pay would be at the ordinary rate fixed for a lieutenant in the R.A.M.C. This is the order of events which will happen in regard to any practitioner whose age brings him within the terms of the Act and who does not apply for exemption before June 24 next.

A second possible course is to apply for exemption, the application being sent to the appropriate local Tribunal. All the same, unless the request is urged on conscientious objections to military service, it will not be judged by this authority. A special clause in the new Act decides that the local Tribunal shall send all applications for exemption submitted by medical men—excepting always those which allege grounds of conscience—to a duly appointed " professional committee." Before such committee the applicant may submit whatever statement and evidence he may think appropriate, but he will be obliged, whatever be the committee's decision, to accept it. As a matter of form the recommendation of the professional committee will be sent to the local Tribunal and the latter has no choice but to adopt it. From such a decision, further, there is no appeal. Now the professional committee formally recognised for England and Wales is the Central Medical War Committee, with, in particular instances, the Committee of Reference appointed by the Royal College of Physicians and the Royal College of Surgeons; and that for Scotland is the Medical Service Emergency Committee. These bodies, in short, take the place of the local Tribunal in judging all claims for exemption when such claims are presented by medical men, though the local Tribunal holds its place if the practitioner's objection to serve is that of the conscientious objector. From this it follows that the fate of the practitioner who claims to be exempt from service—with the single exception just mentioned—lies in the hands of the English (or Scottish) professional committee. Whether the claim is successful or is unsuccessful it will be because of the view taken of it by such committee. If it is unsuccessful there will happen exactly what happens where no claim for exemption is advanced—that is, the practitioner must take service in the Army for the duration of the war and at the ordinary rate of pay for the particular service to which he is commanded.

There is a third course open to the practitioner of military age. It is at once to enrol his name with the appropriate professional committee in England or Scotland as the case may be. But he must take this step without delay, for after June 24 the lists will be closed and the only remaining options will be the two above described. The advantages of enrolment are both to the individual and to the country. First, a practitioner who is in this position has no need to submit to the local Tribunal any claim for exemption which he may wish to urge, and should he by some oversight be called to the Colours a presentation of a certificate of enrolment will at once set him free. That is, a doctor who takes the course here suggested will not be compelled to enter the Army until the professional committee has decided that this is his duty. It is true that enrolment involves an obligation to accept the decision of this committee. But such decision has got to be accepted in any event, for, as already explained, an application for exemption made to a local Tribunal will inevitably be referred to the corresponding professional committee. The only difference is that the practitioner who does not enrol has to go through the formality of an application to the local Tribunal before he reaches the real seat of judgment, while his enrolled fellow avoids the local authority and comes at once to the professional decision. There he may urge whatever reasons he wishes in favour of exemption, and he may perchance gain this in whole or in part. Even if he fails he still is in a better position than the unenrolled practitioner. Called to military service, his term will be for one year and not for the duration of the war, and his pay and allowances will be those fixed for the holder of a temporary commission in the R.A.M.C., and at a somewhat higher level than those granted to the ordinary officer. Thus it is manifest that the individual interest of every practitioner of military age is to enter his name at once with the professional committee appointed in the part of the kingdom in which he resides. The public advantage flowing from prompt enrolment is that it will place the professional committees in a position to consider how best the needs of the Army may be met with a minimum of disturbance to the civil population. The number of men available will be known, as also the manner in which they are distributed in the community. Hence a judgment may promptly be formed as to which practitioners can be advantageously taken for military service and which ought to remain at home. When the figures are known, the determination of the task of each practitioner can be made with confidence. This consideration applies also to men who, though over forty-one years of age, have not reached forty-five. These are eligible for commissions in the R.A.M.C., but they are not under the compulsion of the Military Service Act. Unless a fair proportion of these volunteer satisfactory arrangements for civil practice in view of the needs of the Army are impossible. It is hoped therefore that they will respond to a call which has both a patriotic and a professional sanction.

